# Physicians’ opinions on GBV: a qualitative pilot study in South Africa

**DOI:** 10.3389/fpubh.2026.1716697

**Published:** 2026-09-09

**Authors:** Koustuv Dalal, Swarupa N. Kshirsagar, Corné Davis-Buitendag

**Affiliations:** 1 Division of Public Health Science, Department of Health Sciences, Mid Sweden University, Sundsvall, Sweden; 2 Strategic Communication, University of Johannesburg, Johannesburg, South Africa

**Keywords:** domestic violence, gender based violence, physicians, South Africa, trauma

## Abstract

**Background:**

South Africa has seen a rise in gender-based violence (GBV), which now ranks as a top contributor to years of life lost for women. GBV is a significant public health challenge. Physicians and other medical professionals need targeted knowledge and skills for an adequate response. This study assessed South African physicians’ understanding and opinions on GBV care. It also explored the effects on physicians treating survivors.

**Methods:**

To assess the understanding and opinions of practicing physicians in South Africa, the study used a qualitative method. One mini focus group discussion (mini-FGD) included four physicians from four provinces. This was followed by one in-depth interview (IDI). Researchers conducted thematic analysis using Atlas.ti. They organized themes based on guided prompts.

**Results:**

Physicians identified structural and social barriers like societal issues, inefficient systems, lack of collaborative efforts for GBV, community factors affecting the care, personal financing for GBV care that block survivor access to GBV care. Survivors often return to unsafe environments because of poor support services. Physicians reported stress, fatigue, and helplessness while responding to GBV survivors. While physicians are motivated by empathy to help survivors, they also experience trauma due to the growing number of GBV cases.

**Conclusion:**

The high incidence of GBV in South Africa demands comprehensive attention. Physicians recognized GBV care as a complex socioeconomic and public health issue. They reported that enhanced cooperation between the government and the private sector, with a focus on funding prevention and strengthening infrastructure, would improve care for survivors of GBV.

## Introduction

Gender-based violence (GBV) against women and children is a serious issue. It affects society, communities, legal systems, and public health and is a global challenge (1). It leads to physical, mental, emotional, and social health problems. It also creates a socioeconomic burden as it impacts lives, diminishes a possibility of the survivor living a flourishing and purposeful life and increases institutional burdens, which cost to society. The frequency and severity of GBV varies, creating distinct patterns across the world (2). Globally, 41% of women have experienced either physical, sexual violence, or both from partners (3). GBV happens in many situations across all sociodemographic, economic, religious, and cultural settings (4). GBV survivors are often praised for their silence. They experience numerous effects of violence, including physical injuries (5) and various mental health challenges such as stress, depression, anxiety, and post-traumatic stress disorder (PTSD) (6). These issues often lead to gut health problems (7). Furthermore, survivors may develop physical and social phobias and frequently have suicidal thoughts (8–10). Most importantly, GBV survivors are often forced to assume responsibility for their own care (11) due to lack of support structures, especially in inequitable or unequal settings as per the global dataset (12).

Sustainable Development Goal (SDG) 5 - for Gender Equality highlights elimination of GBV as target 5.2: “Eliminate all forms of violence against all women and girls in public and private life, including trafficking, sexual, and other types of exploitation”. SDG 16 for Peace, Justice, and Strong Institutions targets 16.2: “End abuse, exploitation, trafficking, and all forms of violence against and torture of children” (13).

### GBV and healthcare professionals

As a social phenomenon, GBV is a growing public health concern. Healthcare professionals can play a crucial role by identifying cases, providing essential services, and offering support to survivors and they are recommended to maintain a non-judgmental approach when caring for survivors (14, 15). WHO guidelines suggest that healthcare services need to be appropriate and acceptable, and that providers receive skills and capacity building (14) for the provision of suitable care to GBV survivors. They need more in-depth and specific knowledge and skills to respond appropriately (16) and offer support to the individual.

Globally, assessments of providers’ knowledge of GBV vary (17, 18) and is limited despite their recommended role in global guidelines. Capacity building and clear standard operating procedures can increase readiness to manage GBV (19). In Uganda, a study highlighted providers’ awareness of factors such as stigma and lack of support services (20). Healthcare professionals may face burnout and empathy-based stress after their constant interaction with trauma survivors (21).

Physicians are ethically obliged to inform themselves about manifestations of GBV, identify cases, treat physical and psychological consequences of violence and advocate for suitable provisions for women seeking safe places (22, 23). Physician’s role has been criticized to be enabling medicalization of GBV by a study in high income country (22), however, physicians may be extremely instrumental in low resource settings as the effects of GBV are intensified in inequitable or unequal settings, especially in lower or middle income countries.

### South Africa and GBV

South Africa is one of the world’s most unequal countries (12). The population includes 81.4% Black, 8.2% coloured, 7.3% white, and 2.5% Asian/Indian. Black and poor women and children face more violence. South Africa is reported to have femicide five times higher than the world’s average (24). In 2025, the country declared GBV as a national disaster (25). There have been interventions at national and local level to address the GBV, the local interventions seemed to have limited efforts (24) as the study notes that there are no sustainable community level programming and interventions aimed at changing social norms perpetuating GBV (24). Another study conducted with NGOs in South Africa shows that their efforts are limited to secondary and tertiary approaches to address GBV, there is little focus on primary interventions to prevent the occurrence of GBV (26). The researchers note that there are limited studies (for example (27–29) that explore GBV from perspectives of healthcare providers). Given South Africa’s high prevalence of GBV, resource scarcity and requirement of a multisectoral approach to understand the mechanisms and plan effective interventions, it is important to understand healthcare providers’ perspective and opinions on GBV.

Physicians’ understanding is crucial in combating GBV as they are usually given guidelines to work within a guided framework to address GBV in clinical framework with also supporting clients. This study thus aims to assess South African physicians’ perceptions of GBV care and to explore the effects on physicians who treat survivors of GBV.

## Methods

### Study design

The study employed a qualitative multi-method approach to assess South African physicians’ perceptions of GBV care and to explore the effects on physicians who treat survivors of GBV. (the COREQ checklist is included in the appendix). Recent literature encourages descriptive, qualitative, multi-method approach for gaining in-depth understanding (30, 31). Respondents were purposively selected as the aim required participants to have experience in treating GBV survivors. A mini focus group discussion (mini-FGD) took place with four physicians from various provinces. Then, an in-depth interview (IDI) was held with a senior physician experienced in caring for GBV survivors. The total interviewed sample size consists of 5 physicians.

### Participants and recruitment

Researchers first sent a general email to ten prospective physicians who were known for supporting GBV survivors within selected networks. Network coordinators helped find the best representative physicians from each city and physicians with less than 10 years of experience treating GBV survivors were excluded. Four physicians willing to participate sent their consent. They were all females, aged between 50 and 60 years. Participant 1 (P1) practiced in East London (Eastern Cape), P2 in Cape Town (Western Cape), P3 in Durban (KwaZulu-Natal), P4 in Bloemfontein (Free State). These provinces are diverse with urban–rural population, economic activities and cultural variations.

Based on mini FGD experience, one in-depth interview was conducted with a senior physician, practicing with GBV survivors. The in-depth interview was conducted with another physician (female, age between 50 and 60 years) in Johannesburg, Gauteng, who is respected for her work with GBV survivors *(P5).* The study adhered strictly to the guidelines of the Ethical Committee.

### Data collection procedure

Using mini FGD and in-depth interviews, data was collected through online meeting. The participants were contacted, briefed on the study’s aims, procedures, and ethical issues, including their right to withdraw and were then sent a consent form for online FGD. They read the objectives and online interview guidelines, agreed to participate, and were informed of ethical issues. Two experienced senior researchers led the mini-FGD of four physicians *(P1, P2, P3, P4)*. A unanimous date and time were set for the mini-FGD (32). The session lasted 55 min and was audio recorded for transcription. The most senior researcher took notes.

Based on prompts, notes, early findings, and mini-FGD experience, researchers held a further in-depth interview (IDI) (30, 31) with a physician in Johannesburg, Gauteng, who is respected for her work with GBV survivors *(P5)*. The interview was recorded and fully transcribed. The IDI lasted for 38 min. Data saturation was obtained with in-depth interview with P5 (17).

Trustworthiness in qualitative studies is of paramount importance, and this, along with ethical standards, was achieved in this study by employing Lincoln and Guba’s model, which satisfies the four key essential criteria for ensuring the quality of qualitative research: credibility, confirmability, dependability, and transferability (20, 21). Trustworthy qualitative research outcomes are essential for informing policymakers to improve service provisions and make informed preventive decisions. This was ensured by sharing findings with the participants to ensure there was no overinterpretation.

### Ethical considerations

The study followed the stipulated guidelines based on the ethical committee’s rules and regulations and the World Health Organization guidelines on research on violence against women and children. According to the guidelines of the ethical committees, written informed consent was obtained from each participant (physician) before initiating discussions or interviews. They were informed that the interviews would be recorded, transcribed, anonymized, and then analyzed.

Since the network of physicians dealing with GBV cases in study region is small and well-connected, divulging any specific details (like exact age, smaller area of practice, specialty) risks identification of the participants, compromising the integrity of the study. The study, thus, avoids stating specific demographic details in usual practice of tabular format to evade the risk of identifiability of the participants. Before interviews (mini-FGD & IDI) the participants were clearly informed that if they need any further support for any mental distress after our meeting, necessary support will be provided through psychological counseling and were encouraged to contact the researchers if such situation arises. Ethical clearance for the research was obtained from the Faculty of Humanities Ethics Committee, with ethical clearance number REC-02-151-2022.

### Data analysis

The transcripts were anonymized before analysis. The recordings were deleted from the recording device after transcription. They were then entered into QDA software, Atlas.ti. The study used thematic content analysis to examine the qualitative data (33). An independent coder handled coding, categorization, and thematic creation in Atlas.ti 24 (33, 34). The popular QDA software has been used in several qualitative studies to assist with coding and analysis (33, 35), which helped the research team to work efficiently (33, 36). The prompts provided by the researchers shaped the contents of the themes for physicians, identifying seven sub-themes under the sampled stratum of physicians. Each category contains specific codes associated with its relevant quotations, the independent coder generated summaries for each category, which are available in the project unit. The independent coder performed a light edit of the software-generated summaries to ensure the accuracy of the data and to supplement when some codes appeared not to have been reported. However, substantively, these are software-generated summaries intrinsically linked to the project unit and not in any open domain. The two experienced researchers have controlled the quality in each stage to ensure trustworthiness by sharing data and interpretations with participants.

The codes led to sub-themes which were categorized into two major themes aligning with the objectives of the study. The first category focused on the first objective of physicians’ perception of GBV, which included structural inequalities and barriers to GBV care in South Africa. A category aligning with the aim of the study was used to group sub-themes that indicate societal structures, inequalities, and barriers to GBV care and was arranged based on ecological framework.

The second aim was to explore the effects of treating GBV survivors on physicians. While analyzing, the second category was reiterated to accommodate the observed perpetuation of GBV due to systemic inefficiencies and to include effects on GBV survivors. These sub-themes related to effects of GBV on survivors, including the perpetual nature due to the insufficient support and effects on physicians were categorized as consequences of GBV. The two themes were further presented in a diagram to present the link between them, as identified during the enquiry.

## Results

**Figure 1:**
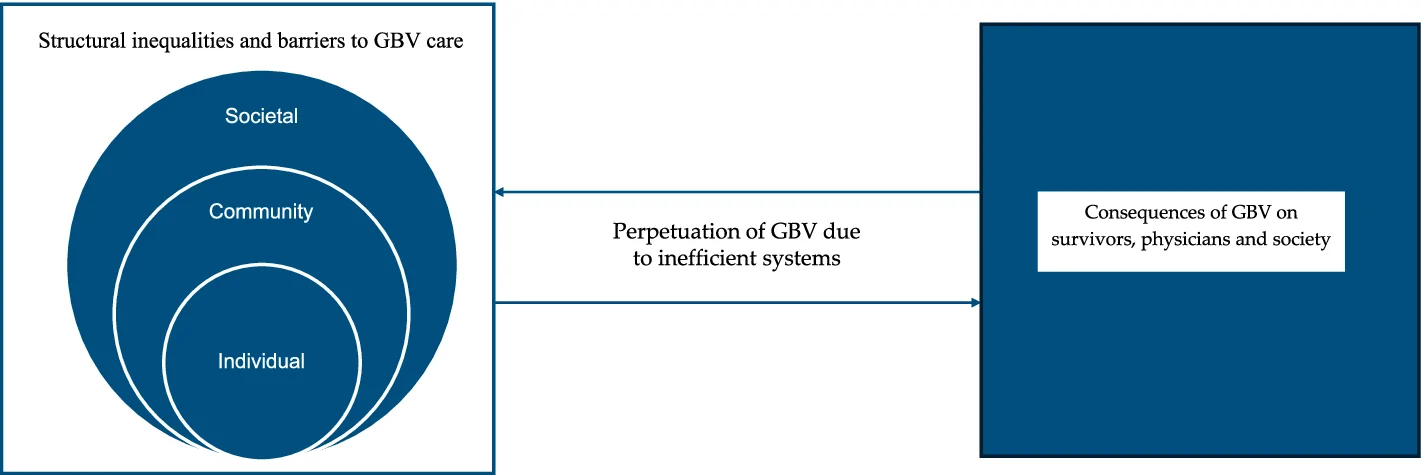
Conceptual framework.

All respondents for the mini-FGDs and IDIs were women physicians with at least ten years of experience and who privately practiced in their respective cities. The first category presented sub-themes of structural inequalities and barriers to GBV care from the physicians’ perspective. The second category presents human and professional consequences of GBV on survivors and physicians. The subthemes of the first category are presented according to the levels of the ecological model of GBV; however, they are depicted as factors affecting GBV care rather than as origins of GBV, as viewed from a care professional’s perspective (Table 1). The interpersonal level was not addressed and is therefore eliminated from the framework. The following figure, based on ecological framework, illustrates the relationship between the themes (Figure 1).

**Table 1:** Thematic representation.

Themes	Sub-themes (as per ecological framework)	
Structural inequalities and barriers to GBV care	Societal issues	
	Inefficient systems	
	Lack of collaborative efforts for GBV	
	Community factors affecting the care	
	Personal financing for GBV care	
Human and professional consequences of GBV	Consequences for survivors	Physical and Mental Health Effects of GBV for survivors
		Trauma and perpetuation of GBV
	Consequences for physicians	Stress and fatigue
		Feelings of powerlessness and frustration

### Structural inequalities and barriers to GBV care

#### Societal issues

Physicians opined that factors like poverty, patriarchy, and policy failures worsen gender-based violence in society. The participants emphasized the importance of addressing food insecurity as a compounding factor that triggers GBV and exacerbates vulnerabilities. Food-insecure households are particularly vulnerable to GBV, as reported cases of abuse may lead to the absence of the mother and further food deprivation of her children in the patriarchal context of society. They discussed escalating issues like unemployment and substance abuse, which were believed to contribute to increased GBV. Specifically at the societal level, physicians noted that the provision of food packets and basic needs assistance tends to inadvertently divert resources and attention away from addressing hidden or under-reported cases of gender based violence. They observed that such survival struggles tend to be aggravated and have a prolonged impact of GBV, especially among the poorest populations. The conversation touched on the historical context of social issues in South Africa. Physicians believe that their current society is stressed, with intergenerational trauma and a deteriorating social fabric.

*“Limited resources are used for food and basic needs. This undermines the actual scenario of violence. The survivors have the survival struggle after which comes their victimization…” (P3).*

#### Inefficient systems

The participants noted that the current support and care systems for GBV were not improving, as there are limited options around support and rehabilitation for survivors. They believed the systems were stagnant and were not addressing GBV care holistically. The DNA kits essential for legal proceedings of sexual abuse were deficient, meaning incomplete provision of medical proof of sexual abuse, leading to subpar support. On the other end of the spectrum of care services, the rehabilitation centers are severely lacking in numbers and quality of support to offer GBV survivors.

*“…DNA is the most important for convicting the perpetrator. We cannot provide any support to the survivors and the judicial system due to lack of DNA kits. The perpetrator knows it. It could lead them to be more desperate and perpetual offenders….” (P2).*

*“There are very few support centres for the GBV survivors, run by civil societies. The overall system lacks a plan and resources...” (P4).*

#### Lack of collaborative efforts for GBV

The physicians discussed the lack of prevention strategies for GBV. The participants believed that GBV has become a generational issue that is affecting the younger generation, and it could be prevented through educational and awareness programs in schools. They emphasized that acknowledging the scourge of violence and normalizing conversations about prevention should be the main components. They thought a generational shift toward gender equality was needed in society. The respondents emphasized the gaping need for support and structural changes to address the current stress on the systems due to GBV. Collaboration across sectors was considered crucial to addressing the existing challenges of stagnant systems and limited resources. They believed this would help with the equitable use of unequal resources.

*“The private sector can come forward and support GBV survivors under their enhanced CSR plan.” (P4).*

#### Community factors affecting healthcare access of GBV survivors

The physicians believed that there was severe underreporting of sexual abuse. Physicians noted a pattern of care-seeking behavior in which poor people access government healthcare facilities, but the lack of infrastructure and other resources can provide only scant evidence. This is further layered by stigma, which makes it difficult for an individual to access care in these facilities. They noted that this also leads to disruption in the continuum of care after first visits. They expressed fear that many poorer communities could not access any healthcare facilities despite the severe burden of GBV. Physicians, who practice privately, stated that they have only a partial view of the issue, as private healthcare clinics primarily serve high- and middle-income patients, offering limited visibility into GBV. They opined that the issue could be much worse than what they know as many poor patients access government health facilities.

*“What we see is tip of the iceberg. A very few GBV survivors visit us. Stigma is the greatest challenge for them…” (P3).*

*“We are in private clinics. It is expensive. Do you think a poor victim can visit here? There are both economic and social barriers especially stigma…” (P2).*

#### Personal financing for GBV care

On an individual level, poverty was a factor which participants believed was a key barrier to care. While the services like post-exposure prophylaxis and counseling were in place, physicians observed that these were expected to be paid out of pocket by the survivor, making it a catastrophic expenditure for the poor. Physicians sympathized with the poorer survivors, recounting that personal financing is a major barrier that affects uptake of support services.

*“The victim must pay for the medical treatments, medicines, and rehabilitation costs if she can afford. Mostly it (GBV care) needs further and repeated visits to healthcare facilities….” (P5).*

*“…One day a girl who was severely sexually abused by her close family member visited me. The girl was so poor, even she or her mother had no money to buy painkiller. Forget about decent food and (other) necessary medicines (or treatments)…” (P1)*

### Human and professional consequences of GBV

#### Consequences for survivors

##### Physical and mental health effects of GBV for survivors

While the question prompted the physical effects visible to the practitioners, the participants, in fact, devoted many of their responses to recalling the mental health effects of GBV. GBV was noted to have significant impacts on survivors through mental health issues, trauma, and physical illnesses like hypertension. There were specific details of the extreme difficulties faced by rape survivors, as there were ongoing follow-up actions needed, including the possibilities of pregnancy.

##### Trauma and perpetuation of GBV

The physicians indicated elevated levels of trauma in the survivors and described it as compounding traumas. They explained that a survivor faces multiple concerns after she seeks care for GBV. A chief concern is being separated from children, food security, and self-care/survival after the abuse. Society and family tend to support a mother more than a single woman, which might be a risky situation, pushing them further into social exclusion. This, they believed, added to the survivor’s vulnerability for repeated encounters with GBV. They also noted that those women who were not mothers are less supported in the community, being left with very few options for support structures.

The participants noted that the current support and care systems for GBV were not improving, as there are limited options around support and rehabilitation for survivors. This often leads survivors to return to the same place where GBV happened to them, and they might continue to suffer with multiple abuses, especially when the perpetrator is an intimate partner or a close family member. They believed the lack of resources for critical services like shelters, police, and prosecutors led to a harmful and damaging perpetuation of violence and poverty, with the system not moving in an optimistic direction for GBV support.

*“It is difficult for the GBV survivors to get necessary supports from the society and family except mothers. It might cause more dangerous situations for the survivors. They become more traumatized and feel helpless. ……… There is a spiral negative effect on the GBV survivors in terms of trauma, helplessness, frustration, and extreme social exclusion………” (P5).*

*“…After (my) treatment, one of my patients had to go back to the same house where she would live with the same person (the perpetrator)….” (P1).*

#### Consequences for the physicians

##### Stress and fatigue

The physicians acknowledged that they often experience secondary trauma due to the nature of their work and the frequency of meeting GBV survivors. Among such situations, meeting younger survivors was stated to be extremely disturbing and sad for the participants. There are a few support services for their secondary trauma. The IDI participant highlighted the emotional toll of having to witness their suffering.

*“I cannot tolerate. Most of the day, there are at least one such GBV survivors, sexual violence. Can you imagine they are as young as eight-nine years? I cannot eat that evening, and it has longstanding effects on me….” (P5).*

Simultaneously, the work stress was also high as dealing with GBV cases means a lot of collaborations with psychologists and social workers to support survivors. There was a report of suicide because of the scale of GBV and the trauma. The mini-FGD participants mentioned that they themselves could not get enough mental health support and had financial constraints to access these services. Despite the struggles, they reported finding comfort in offering kindness and hope to those in need, especially among marginalized communities.

##### Feelings of powerlessness and frustration

They discussed feelings of powerlessness and frustration due to difficulties in providing services, navigating bureaucratic hurdles within the prison system, and facing challenges with follow-through from authorities, particularly at police stations. They felt that the positive evolutions in the social service responses are not foreseeable, even in the medium term. They expressed anger at the treatment of patients seeking help.

They also highlighted that apart from physicians, the shelter staff that deals with GBV survivors daily share the same feelings of helplessness and fatigue. They also indicated that all organizations cannot afford counseling or supervision for their staff. The fatigue was believed to originate from dealing with similar types of GBV cases over an extended period.

*“The shelter staff are also helpless. They are also traumatized day after day, night after night by dealing with the same type of GBV survivors.” (P5).*

## Discussion

The study aimed to assess perceptions of physicians in South Africa regarding GBV care and explore the effects on physicians for treating GBV survivors. The factors are discussed from a healthcare professional’s point of view who met survivors after the GBV incidence and witnessed the survivor’s journey with the system, these factors are structured into the levels of the ecological model (37), from societal to individual levels. Some of the risk factors and origins of GBV in South Africa’s society were discussed by physicians, along with how the health and other auxiliary systems work for GBV survivors. In addition to this, the current study inquired about the effects of GBV on physicians and potential solutions. The second category speaks about human and professional consequences of GBV.

### Factors on various levels of the ecological model affecting GBV care

The study identified numerous factors at various levels that affect care delivery and influence GBV care-seeking. On societal levels, participants acknowledged the existence of patriarchy, poverty, and policy failures as key risk factors for GBV. Physicians pointed out food insecurity, a less prominent aspect of GBV, which has recently been highlighted as both a direct and indirect factor in the incidence of GBV (38, 39). Food insecurity was viewed as a cause of diverting resources from care services, as food packet distribution was prioritized, leading to insufficient care provision. This highlights the inequality and poverty in the South African society, pointing to GBV being a multifaceted issue and having consequences on the societal level. On the societal level, the inefficiency of systems was discussed at length, pointing toward the necessity of a multisectoral approach for GBV care (38). Collaborative efforts between multiple actors are possible if the private sector is considered for supporting infrastructure, by ensuring the services as suggested in guidelines for integrating GBV intervention (39). The physicians pointed out the requirement of the spectrum of services from the initial step of using DNA kits to prosecute the perpetrator to the last step of rehabilitation of the survivors, which in the current scenario is insufficient, considering the incidence of GBV.

According to physicians, beliefs played a significant role in the lack of care-seeking. The commonly perceived stigma around GBV hinders care-seeking behavior (40, 41), mostly due to the fear of social exclusion, and a fear of being subjected to discrimination due to having been subjected to sexual violence. This is bound to affect care-seeking behavior, especially mental health care-seeking, as found by a Ugandan study (42). Earlier studies have found that only about 7% women who experienced GBV reported to formal sources (43), which concurs with the physicians’ opinion about underreporting. This awareness among physicians about underreporting is important and can be leveraged to inform policy development and facilitate a more thorough investigation of the issue. Earlier research shows that training and capacity-building programs for physicians on universal screening of GBV are implemented and often cited as best practices in some guidelines (22). While such programs have been assessed to have limited effects on reducing IPV (44), they may be helpful in high-incidence settings to increase awareness of the issue.

On an individual level, poverty and out-of-pocket expenditure played a crucial role in care seeking as a barrier from the physicians’ perspective. It is implied that poorer people do not access private clinics, leaving them with only the option of government healthcare services. However, an economic evaluation found that in 2021, the total out-of-pocket expenditure for GBV survivors was approximately R10 billion (45), indicating possible catastrophic expenditure. The personal financing for GBV care is also a contributing factor to the perpetual poverty of survivors and highlights the need for survivors’ empowerment.

### Interplay of systemic inefficiency and perpetuation of violence

The study found an interesting link between systemic inefficiency and consequences of GBV that a survivor may be pushed into a perpetual cycle of GBV due to inadequate systemic support and inequality of resources, unable to access suitable care, and returning to the place of GBV. This is a significant finding of the study, as it is presented from the perspective of care professionals, highlighting the importance of providing appropriate support and rehabilitation services for GBV.

Participants discussed the socioeconomic and administrative aspects that accentuate the effects of GBV. DNA testing equipment is a basic requirement for providing medicolegal support in GBV cases. The administration’s inability to ensure the availability of such testing kits was a significant bottleneck, suggesting that, despite the high incidence of GBV, essential services related to it have not been prioritized by the system. As is known, this is not only limited to the unavailability of DNA kits but also reflects in the lack of other rehabilitative and support services like shelters, police, and prosecutors for GBV survivors. This points to the administrative apathy about GBV and is a known risk to perpetuating the abuse cycle (37, 46) Underreporting of the issue, as mentioned by physicians, could also stem from the same issue in government setups, as essential diagnostics or tests are not available, creating a systemic barrier for service seekers and pushing them away from GBV care.

Participants perceived South Africa as a stressed society and indicated GBV as a generational issue, advocating for community efforts to address the same. An earlier study has noted wife-beating attitudes among younger generations of men and women across African countries (47), which validates the concerns raised by participants in our study, highlighting the need for urgent prevention strategies in South African society. Multiple studies have highlighted the interaction between patriarchy, poverty, the historical context of these social issues and GBV in the region (48, 49).

### Consequences for the professionals

The physicians shared the traumatic feeling after treating GBV survivors. They also pointed out that seeing younger GBV survivors was hard. Earlier investigations suggest that existing healthcare professionals should expand their roles and assume functions typically performed by other service professionals (50). This is bound to increase the stress of caregivers among physicians. The lack of support for healthcare professionals is a known factor in burnout, especially in care related to GBV (51). A Nigerian study advocates for formal skill training for medical students and strongly recommends a review of the medical curriculum to accommodate the needs of GBV care (52). Similar on-the-job training may be beneficial for currently practicing physicians in South Africa. Empathy and kindness were found to be a driving force for the participants, where they find comfort in helping people, especially those who are marginalized. These are unique findings highlighting providers’ experiences in a few cities in South Africa regarding GBV survivors, as far as our knowledge goes. These, however, cannot be generalized for all care providers; instead, they may be interpreted as one of the key values to be instilled in physicians and care providers as they deliver services to GBV survivors.

### Strengths and limitations

The study employed qualitative methods to gain an in-depth understanding of physicians’ opinions on GBV, which is crucial for policymaking. Mini-FGD offered the advantages of generating specific ideas across the physicians’ group interaction. It helped to achieve in-depth, qualitative understandings by allowing for non-verbal cues and dynamic group discussions. The researchers implemented strategies to eliminate the risk of participant bias and dominance during the online mini-FGD. Considering the travel and physical meeting costs, time constraints, and sensitive topic issues, the online mini-FGD emerged as the most suitable method in the study context. Using mini-FGD of physicians of four regions first and then IDI of a very experienced physician in the capital city provided a rich and in-depth appreciation of the physicians in the current study context by providing and revealing the “how” and “why” behind GBV issues, primarily to receive a more inclusive and robust qualitative perception (30, 31). The data saturation was confirmed.

The small sample size and qualitative nature of the study should be considered when generalizing the findings. The awareness level of physicians across South Africa will vary, and the intricacies of the issue may not be fully understood by everyone within the system. Similarly, not every physician might lead the way with kindness and empathy as the participants showed. There are differences in the healthcare service delivery at private and government healthcare facilities. Since the respondents are all privately practicing physicians, they may have certain biases toward the service delivery at the government facilities. Thus, the findings must be understood as a reflection of care providers and further interpreted to build a way forward in addressing the rising issue of GBV.

It must be elucidated here that even though the current study used QDA software (Atlas.ti) based on AI, the analysis was chiefly conducted by two experienced researchers. An independent coder was used to generate the summaries to be used further for theming. However, the summaries were crosschecked with the content, and the coder was prompted to do editing for accuracy of data or to supplement when some codes seemed not to be reported on after the human review. Further thematic analysis was undertaken by the research team along with continuous validation of the analysis by the experienced researchers. This type of analysis using Web QDA has been confirmed by literature for structured coding of qualitative data that facilitates efficient data organization and management by ensuring systematization and transparency throughout the analytical process (53).

## Conclusion

The study brings forth healthcare professionals’ perspectives on the recently relevant issue of GBV in South Africa. The study highlights the link between inefficient systems and the perpetuation of GBV as perceived by healthcare professionals, along with the consequences of GBV care provision on the physicians.

The South African government has recently recognized GBV as a national disaster. Acknowledgment is the first step, and adopting a lifecycle approach with prevention strategies can have a lasting impact on reducing GBV prevalence. Strengthening the collaborative effort should be a key step, considering the magnitude of the issue and the requirement for resources. Bringing males into the strategies and at the centre of educational and awareness programs for prevention strategies should also be considered. Stress and fatigue among physicians are significant workplace health issues that can lead to severe burnout if left unaddressed. This should be considered one of the key factors in planning GBV care services in an area, to prevent caregivers’ stress from building up among healthcare professionals. Adequate mental health support for both survivors and care professionals should be prioritized to work as empowering and preventive.

## Data Availability

The datasets presented in this article are not readily available because according to Ethical Committe’s guideline, we are not able to share the data. Requests to access the datasets should be directed to koustuv.dalal@miun.se.

## References

[1] Jewkes R Abrahams N. Global burden of disease from intimate partner violence against women and sexual violence against children: a call to action. Lancet. (2026) 407:2–3. doi: 10.1016/S0140-6736(25)02598-X, 41483900

[2] Davis-Buitendag C Chakraborty S Dalal K. Where, when and what? A national support Centre scenario for victims of gender-based violence. Discov Soc Sci Health. (2025) 5:53. doi: 10.1007/s44155-025-00203-7

[3] Dalal K Biswas A Chakraborty S Davis C. Prevalence, socioeconomic determinants, and regional disparities of intimate partner violence (IPV) in India: a review over the three National Family Health Surveys. Shiraz E-Med J. (2025) 26. doi: 10.5812/semj-148693

[4] Breiding M. Basile K. C. Smith S. G. Black M. C. Mahendra R. R. Intimate partner violence surveillance: uniform definitions and recommended data elements. Version 2.0 (2015). Available online at: https://stacks.cdc.gov (Accessed September 2, 2025).

[5] Leslie KT Matshidza ST Aluko O. Musculoskeletal injuries from gender-based violence at a tertiary hospital orthopaedic Centre, Central South Africa. Injury. (2025) 56:112061. doi: 10.1016/j.injury.2024.112061, 39615309

[6] Stein C Flor LS Gil GF Khalil M Herbert M Aravkin AY . The health effects associated with physical, sexual and psychological gender-based violence against men and women: a burden of proof study. Nat Hum Behav. (2025) 9:1201–16. doi: 10.1038/s41562-025-02144-2, 40210704 PMC12185316

[7] Perona M Benasayag R Perelló A Santos J Zárate N Zárate P . Prevalence of functional gastrointestinal disorders in women who report domestic violence to the police. Clin Gastroenterol Hepatol. (2005) 3:436–41. doi: 10.1016/s1542-3565(04)00776-1, 15880312

[8] Kalokhe A Del Rio C Dunkle K Stephenson R Metheny N Paranjape A . Domestic violence against women in India: a systematic review of a decade of quantitative studies. Glob Public Health. (2017) 12:498–513. doi: 10.1080/17441692.2015.1119293 PMC4988937 26886155

[9] Kaur R Garg S. Addressing domestic violence against women: an unfinished agenda. Indian J Community Med. (2008) 33:73–6. doi: 10.4103/0970-0218.40871 PMC2784629 19967027

[10] Lutgendorf MA. Intimate partner violence and women’s health. Obstet Gynecol. (2019) 134:470–80. doi: 10.1097/AOG.0000000000003326, 31403968

[11] Joyner K Rees K Honikman S. Intimate partner violence (IPV) in South Africa: How to break the vicious cycle. (2015). Available online at: https://pmhp.za.org/wp-content/uploads/IPV_policybrief.pdf (Accessed September 2, 2025).

[12] Johnson NL Benner M Lipp NS Siepser CF Rizvi Z Lin Z . Gender inequality: a worldwide correlate of intimate partner violence. Women's Stud Int Forum. (2024) 107:103016. doi: 10.1016/j.wsif.2024.103016, 42574925

[13] South Africa Most Unequal Country in the World: Report | International Center for Transitional Justice. (2022). Available online at: https://www.ictj.org/node/35024 (Accessed September 2, 2025).

[14] World Health Organization (WHO). United Nations Population Fund (UNFPA), United Nations High Commissioner for Refugees (UNHCR). Clinical management of rape and intimate partner violence survivors: developing protocols for use in humanitarian settings. Geneva: World Health Organization; 2020. Licence: CC BY-NC-SA 3.0 IGO. Available online at: https://iris.who.int/server/api/core/bitstreams/13f3761a-5518-4b27-92d6-a5e6c7aa283e/content (Accessed September 2, 2025).

[15] Ali P. Gender-based violence and the role of healthcare professionals. Nurs Open. (2017) 5:4–5. doi: 10.1002/nop2.120, 29344388 PMC5762759

[16] World Health Organization. Responding to intimate partner violence and sexual violence against women (2013). Available online at: https://www.who.int/publications/i/item/9789241548595 (Accessed September 2, 2025). 24354041

[17] Mtaita C Safary E Simwanza K Mpembeni R Likindikoki S Jahn A. Knowledge, implementation, and gaps of gender-based violence management guidelines among health care workers. Int J Environ Res Public Health. (2023) 20:20. doi: 10.3390/ijerph20075409, 37048027 PMC10093802

[18] Colombini M Mayhew SH García-Moreno C d’Oliveira AF Feder G Bacchus LJ. Improving health system readiness to address violence against women and girls: a conceptual framework. BMC Health Serv Res. (2022) 22:1429. doi: 10.1186/s12913-022-08826-1, 36443825 PMC9703415

[19] Teshome L Adugna H Deribe L. Health providers readiness in managing intimate partner violence in public health institutions, Ethiopia. PLoS One. (2023) 18:e0295494. doi: 10.1371/journal.pone.0295494, 38134007 PMC10745191

[20] Anguzu R Cassidy LD Nakimuli AO Kansiime J Babikako HM Beyer KMM . Healthcare provider experiences interacting with survivors of intimate partner violence: a qualitative study to inform survivor-centered approaches. BMC Womens Health. (2023) 23:584. doi: 10.1186/s12905-023-02700-w, 37940914 PMC10634177

[21] Chau CW Norton C Kruzan KP Jacobs M. “All day, every day, listening to trauma”: investigating features of digital interventions for empathy-based stress and burnout. Proc SIGCHI Conf Hum Factor Comput Syst. (2025) 2025:374. doi: 10.1145/3706598.3713588, 41988484 PMC13077721

[22] Cavanagh A Kimber M MacMillan HL Ritz SA Vanstone M. Conceptualizing physicians’ roles in addressing intimate partner violence: a critical discourse analysis of resources for Canadian physicians. Violence Against Women. (2023) 29:1640–69. doi: 10.1177/10778012221114922, 35989661 PMC10248313

[23] Schmuel E Schenker JG. Violence against women: the physician’s role. Eur J Obstet Gynecol Reprod Biol. (1998) 80:239–45. doi: 10.1016/s0301-2115(98)00140-7, 9846677

[24] Mkwananzi S Nathane-Taulela M. Gender-based violence and femicide interventions-perspectives from community members and activists in South Africa. Front Glob Womens Health. (2024) 5:1199743. doi: 10.3389/fgwh.2024.1199743, 39113900 PMC11303172

[25] Mosebo T. Gender based violence (GBV) in South Africa: a National Disaster!. (2025) Available online at: https://www.gicj.org/topics/thematic-issues/women-gender-equality/4322-gender-based-violence-gbv-in-south-africa-a-national-disaster (Accessed September 2, 2025).

[26] Kieser J Duffy CG. Unveiling the challenges of gender-based violence NGOs in South Africa. Violence Against Women. (2026) 32:640–65. doi: 10.1177/10778012251348425, 40495601 PMC12664913

[27] Mohokare FJ Tlalajoe-Mokhatla N. Managing gender-based violence patients: implications for the practices and attitudes of emergency medical care providers. Pan Afr Med J. (2024) 49:108. doi: 10.11604/pamj.2024.49.108.38454, 40093339 PMC11907716

[28] Artz L Meer T Aschman G. Legal duties, professional obligations or notional guidelines? Screening, treatment and referral of domestic violence cases in primary health care settings in South Africa. African J Primary Health Care Family Med. (2018) 10:e1. doi: 10.4102/phcfm.v10i1.1724, 29943618 PMC6018123

[29] Phaswana-Mafuya RN Phalane E Zungu N Musekiwa A Ramalepe L Bagg K . The health sector response to gender-based violence and sexual reproductive health programs in the commonwealth and selected African countries: protocol for a mixed methods systematic review and Meta-analysis. JMIR Res Protoc. (2025) 14:e67571. doi: 10.2196/67571, 40966689 PMC12491884

[30] Guest G. Namey E. O’Regan A. Godwin C. Taylor J. Comparing Interview and Focus Group Data Collected in Person and Online [Internet]. Washington (DC): Patient-Centered Outcomes Research Institute (PCORI); (2020) 36701499

[31] Rynes SL Bartunek JM. "Qualitative research: it just keeps getting more interesting!". In: Handbook of Qualitative Organizational Research Routledge (2015)

[32] Schulze L Trenz M Cai Z Tan CW. Conducting online focus groups - practical advice for information systems researchers. Commun Assoc Inf Syst. (2023) 52:385–428. doi: 10.17705/1CAIS.05216

[33] Soratto J. Pires DEP de Friese S. Thematic content analysis using ATLAS.Ti software: potentialities for researchs in health. Rev Bras Enferm (2020);73:e20190250. doi:doi: 10.1590/0034-7167-2019-0250 32321144

[34] ATLAS.ti Scientific Software Development GmbH (2023) ATLAS.Ti mac (version 23.2.1) [qualitative data analysis software]Available online at: https://atlasti.com (Accessed September 2, 2025).

[35] Gizaw AT Muktar SA Wondimagegene YA Abayneh M Sefere BZ Tareke KG . Barriers to sexual and reproductive health communication in Southwest Ethiopia: perspectives of parents, youths, and teachers. Front Reprod Health. (2025) 7:1444603. doi: 10.3389/frph.2025.1444603, 40433455 PMC12106455

[36] Li R Che Hassan N Saharuddin N. College student’s academic help-seeking behavior: a systematic literature review. Behav Sci (Basel). (2023) 13:637. doi: 10.3390/bs13080637, 37622777 PMC10451185

[37] Heise LL. Violence against women: an integrated, ecological framework. Violence Against Women. (1998) 4:262–90. doi: 10.1177/1077801298004003002, 12296014

[38] RESPECT women – Preventing violence against women [internet]. Available online at: https://www.who.int/publications/i/item/WHO-RHR-18.19 (Accessed September 2, 2025).

[39] Inter-Agency Standing Committee. (2015). Guidelines for Integrating Gender-Based Violence Interventions in Humanitarian Action: Reducing risk, promoting resilience and aiding recovery. (Accessed September 2, 2025).

[40] Muuo S Muthuri SK Mutua MK McAlpine A Bacchus LJ Ogego H . Barriers and facilitators to care-seeking among survivors of gender-based violence in the Dadaab refugee complex. Sex Reprod Health Matters. (2020) 28:1722404. doi: 10.1080/26410397.2020.1722404, 32075551 PMC7887977

[41] Ngoma-Hazemba A Chavula MP Sichula N Silumbwe A Mweemba O Mweemba M . Exploring the barriers, facilitators, and opportunities to enhance uptake of sexual and reproductive health, HIV and GBV services among adolescent girls and young women in Zambia: a qualitative study. BMC Public Health. (2024) 24:2191. doi: 10.1186/s12889-024-19663-8, 39138556 PMC11321158

[42] Amone-P’Olak K Elklit A Dokkedahl SB. PTSD, mental illness, and care among survivors of sexual violence in northern Uganda: findings from the WAYS study. (2018). Available online at: https://psycnet.apa.org/doiLanding?doi=10.1037%2Ftra0000295 (Accessed September 2, 2025). 10.1037/tra0000295 28758765

[43] Palermo T Bleck J Peterman A. Tip of the iceberg: reporting and gender-based violence in developing countries. Am J Epidemiol. (2014) 179:602–12. doi: 10.1093/aje/kwt295, 24335278 PMC3927971

[44] Feltner C Wallace I Berkman N Kistler CE Middleton JC Barclay C . Screening for intimate partner violence, elder abuse, and abuse of vulnerable adults: evidence report and systematic review for the US preventive services task force. JAMA. (2018) 320:1688–701. doi: 10.1001/jama.2018.13212, 30357304

[45] The Costly Impact of GBV Private Sector Perceptions & Realities in South Africa. GBV-Report22_050822.Pdf. (2022). Available online at: https://www.svai.africa/wp-content/uploads/2022/08/GBV-Report22_050822.pdf (Accessed September 2, 2025).

[46] Govender I. Gender-based violence - an increasing epidemic in South Africa. S Afr Fam Pract. (2023) 65:e1–2. doi: 10.4102/safp.v65i1.5729 PMC10091185 37042525

[47] Choi SYP Ting KF. Wife beating in South Africa: an imbalance theory of resources and power. J Interpers Violence. (2008) 23:834–52. doi: 10.1177/0886260507313951, 18292404

[48] Hatcher AM Page S Aletta Van Eck L Pearson I Fielding-Miller R Mazars C . Systematic review of food insecurity and violence against women and girls: mixed methods findings from low- and middle-income settings. PLoS Glob Public Health. (2022) 2:e0000479. doi: 10.1371/journal.pgph.0000479, 36962559 PMC10021293

[49] Hatcher AM Stöckl H McBride RS Khumalo M Christofides N. Pathways from food insecurity to intimate partner violence perpetration among peri-urban men in South Africa. Am J Prev Med. (2019) 56:765–72. doi: 10.1016/j.amepre.2018.12.013, 30905482

[50] Larsen J Chapman J Armstrong A. Child sexual abuse in KwaZulu-Natal, South Africa. Trans R Soc Trop Med Hyg. (1998) 92:262–4. doi: 10.1016/S0035-9203(98)91001-X, 9861391

[51] Duchesne E Nathoo A Walker M Bartels SA. Patient and provider emergency care experiences related to intimate partner violence: a systematic review of the existing evidence. Trauma Violence Abuse. (2023) 24:2901–21. doi: 10.1177/15248380221118962, 35997064 PMC10594849

[52] Fawole OI van Wyk JM Balogun BO Akinsola O Adejimi A. Preparing medical students to recognize and respond to gender based violence in Nigeria. Afr Health Sci. (2019) 19:1486–98. doi: 10.4314/ahs.v19i1.22, 31148976 PMC6531973

[53] Machado ALG Vieira NFC. Use of webQDA software on qualitative nursing research: an experience report. Rev Bras Enferm. (2020) 73:e20180411. doi: 10.1590/0034-7167-2018-0411, 32267413

